# Whitefly Endosymbionts: Biology, Evolution, and Plant Virus Interactions

**DOI:** 10.3390/insects11110775

**Published:** 2020-11-10

**Authors:** Sharon A. Andreason, Emily A. Shelby, Jeanette B. Moss, Patricia J. Moore, Allen J. Moore, Alvin M. Simmons

**Affiliations:** 1 U.S. Department of Agriculture, Agricultural Research Service, U.S. Vegetable Laboratory, Charleston, SC 29414, USA; sharon.andreason@usda.gov; 2 Department of Entomology, University of Georgia, Athens, GA 30602, USA; eshelby@uga.edu (E.A.S.); jeanette.moss@uga.edu (J.B.M.); pjmoore@uga.edu (P.J.M.); ajmoore@uga.edu (A.J.M.)

**Keywords:** *Bemisia tabaci*, *Portiera*, *Hamiltonella*, bacteriome, symbiotic bacteria, obligate endosymbiont, facultative endosymbiont, *Begomovirus*, GroEL

## Abstract

**Simple Summary:**

Whiteflies feed on plant sap and cause many problems on agricultural crops around the world. Whiteflies have endosymbiotic bacteria in cells inside their bodies that help them to feed on plants. Therefore, the sweetpotato whitefly feeds on a wide range of plants including many vegetable crops, and there are different types of these bacteria which do not all occur in the same whitefly population. In this paper, we focus on endosymbiotic bacteria that are associated with different biotypes of the sweetpotato whitefly with emphasis on their biological characteristics, diversity, and their interactions with whitefly-transmitted plant viruses. This information will be useful to the scientific community for the development of strategies to disrupt these bacteria and provide better whitefly control.

**Abstract:**

Whiteflies (Hemiptera: Aleyrodidae) are sap-feeding global agricultural pests. These piercing-sucking insects have coevolved with intracellular endosymbiotic bacteria that help to supplement their nutrient-poor plant sap diets with essential amino acids and carotenoids. These obligate, primary endosymbionts have been incorporated into specialized organs called bacteriomes where they sometimes coexist with facultative, secondary endosymbionts. All whitefly species harbor the primary endosymbiont *Candidatus* Portiera aleyrodidarum and have a variable number of secondary endosymbionts. The secondary endosymbiont complement harbored by the cryptic whitefly species *Bemisia tabaci* is particularly complex with various assemblages of seven different genera identified to date. In this review, we discuss whitefly associated primary and secondary endosymbionts. We focus on those associated with the notorious *B. tabaci* species complex with emphasis on their biological characteristics and diversity. We also discuss their interactions with phytopathogenic begomoviruses (family *Geminiviridae*), which are transmitted exclusively by *B. tabaci* in a persistent-circulative manner. Unraveling the complex interactions of these endosymbionts with their insect hosts and plant viruses could lead to advancements in whitefly and whitefly transmitted virus management.

## 1. Introduction

Whiteflies (Hemiptera: Aleyrodidae) are globally significant agricultural pests and virus vectors causing direct and indirect damage to crops with estimated losses totaling billions of dollars (US) annually worldwide [1]. The notorious whitefly species *Bemisia tabaci* (Gennadius) is of particular concern due to its highly polyphagous and prolific nature, its composition as a cryptic species, and its transmission of plant viruses [2,3,4]. *Bemisia tabaci* is a natural vector of persistently transmitted begomoviruses (family *Geminiviridae*) as well as some semi-persistently and non-persistently transmitted plant viruses [5]. Other known whitefly vectors include the greenhouse whitefly, *Trialeurodes vaporariorum* Westwood, the bandedwinged whitefly, *Trialeurodes abutilonea* (Haldeman), and the castor bean whitefly, *Trialeurodes ricini* (Misra) [4]. Virus transmission and direct forms of plant damage, such as induction of phytotoxic silvering symptoms, are enabled by whitefly feeding within plant vasculature via piercing-sucking mouthparts.

Sap-feeding insects, including whiteflies, subsist on inferior plant diets enabled by their evolutionary incorporation of endosymbiotic bacteria [6,7]. Obligate intracellular bacteria in whiteflies have directly facilitated their hosts’ adaptation to nutritionally limited phloem diets, which are high in carbohydrates but low in essential amino acids [8,9]. In *B. tabaci* and other whiteflies, the maternally inherited primary (obligate) endosymbiont is *Candidatus* Portiera aleyrodidarum [10]. This endosymbiont is required for the fitness and survival of the insect host. In addition to primary endosymbionts, maternally inherited secondary (facultative) endosymbionts are often found in sap-feeding hosts, and *B. tabaci* harbors highly diverse communities of these endosymbionts [11]. Seven genera of secondary endosymbiotic bacteria are associated with *B. tabaci*, including *Hamiltonella* [12], *Rickettsia* [13], *Wolbachia* [14], *Arsenophonus* [12], *Cardinium* [15], *Fritchea* [16], and *Hemipteriphilus* [17]. These secondary endosymbionts have various roles in whitefly fitness but are not required for host survival, and some are demonstrated to influence virus transmission efficiency [18].

In this review, we discuss the evolution and biology of whitefly endosymbionts and their interactions with plant viruses. We focus on the primary and secondary endosymbionts of the species *B. tabaci*, in which the roles of endosymbionts are complex and complicated by the diversity of secondary endosymbionts and the cryptic nature of the host species. Untangling the intricate relationships between symbiont and host and understanding their roles in plant virus transmission should offer novel insights for whitefly management and virus control.

## 2. Biology of Whitefly Endosymbionts

### 2.1. Classification and Function

To thrive on the nutritionally limited diet of plant phloem, whiteflies, like other piercing-sucking sap-feeding insects, have ubiquitously incorporated and coevolved with obligate, primary endosymbionts [10,19,20]. *Candidatus* Portiera aleyrodidarum (hereafter referred to as *Portiera*), the primary endosymbiont of all whitefly species, provides essential amino acids, carotenoids, and other metabolites that its host is unable to produce on its own and does not receive in its diet or from other symbionts [10,21]. *Portiera* is a gammaproteobacterium with a highly reduced genome (Table 1), a trait which is indicative of the genomic decay that occurs in the coevolution of obligate intracellular symbiotic bacteria with their host [22].

In addition to its primary endosymbiont, *B. tabaci* can also harbor secondary endosymbionts, of which bacteria in seven genera including *Hamiltonella*, *Arsenophonus*, *Rickettsia*, *Wolbachia*, *Cardinium*, *Fritschea*, and *Hemipteriphilus* (Table 1) have been identified. These facultative endosymbionts demonstrate a variety of relationships with their host, ranging from parasitism, which may be highly subject to environmental conditions [24,25], to mutualism, with their products incorporated in host metabolism pathways [26]. Enterobacterium Ca. Hamiltonella defensa (hereafter *Hamiltonella*) may be more the latter type given its involvement in essential amino acid biosynthesis pathways and production of B vitamins [26,27]. However, it is also suggested to be a nutritional parasite competing with *Portiera* for host derived resources [25]. *Arsenophonus*, another enterobacterium, may also have a more mutualistic type of relationship with *B. tabaci* as it too is suggested to help provide essential nutrients [12,28]. Both *Hamiltonella* and *Arsenophonus* appear to be fixed within *B. tabaci* populations [28]. In contrast, the alphaproteobacteria *Rickettsia* and *Wolbachia*, class Rickettsiales, are generally not fixed in populations and may represent more dynamic, parasitic-like associations with *B. tabaci* [13,14,29]. Two groups of the genetically diverse *Rickettsia* genus have been found associated with *B. tabaci*: *Rickettsia* Bellii, which includes many pathogenic strains [30,31,32], and, recently, *Rickettsia* Torix [23]. Another alphaproteobacterium in the class Rickettsiales that is not fixed in *B. tabaci* populations, Ca. Hemipteriphilus asiaticus (hereafter *Hemipteriphilus*), is closely related to a mite-borne human pathogen in the genus *Orientia* [17,33]. *Hemipteriphilus* so far appears to be restricted to the bacteriocytes, but its function and effect on host fitness is unknown. *Candidatus* Cardinium hertigii (Bacteroidetes) has a reduced and dynamic genome that lacks robust cofactor and amino acid biosynthesis capability and, therefore, is not suspected to be important to host nutrition metabolism [15,34]. The final known secondary endosymbiont, Ca. Fritschea bemisiae (hereafter *Fritschea*) (Parachlamydiales), occurs in the bacteriocytes, but little is known about its function in *B. tabaci* [16]. Altogether, much remains to be elucidated about the specific functions of secondary endosymbionts, especially when considering the diverse makeup of endosymbiont communities in *B. tabaci*.

### 2.2. Morphology, Localization, and Transmission

Observations of the structure of whitefly bacteriomes, also called mycetomes, and endosymbionts began with comparing those of *B. tabaci* and *T. vaporariorum* [35]. Bacteriomes, which are yellow-orange in color and easily observable in late immature stages, are specialized organs composed of large cells referred to as bacteriocytes, or mycetocytes, which house intracellular endosymbiotic bacteria [19,35]. Whitefly bacteriocytes were observed to contain large nuclei (sometimes multi-nucleate), mitochondria, ribosomes, and granular bodies, along with prokaryotic organisms of two described types, pleomorphic and coccoid [35]. While the structure of the more abundant pleomorphic type was observed to lack a distinct cell wall in both species, that of the coccoid type was different, with a much thicker cell wall in *T. vaporariorum*. These results were supportive of earlier 16S rDNA sequencing of whitefly bacteriomes [36] suggesting two endosymbiotic gram-negative eubacteria in *B. tabaci*. A follow up study comparing populations of *B. tabaci* MEAM1 (Middle East Asia Minor 1; previously, B biotype/*B. argentifolii*,) and NW (New World) group whiteflies (including A biotype and Jatropha biotype populations; see [37,38]) demonstrated differences in the cryptic groups [39]. *Bemisia tabaci* MEAM1 appeared as observed in Costa et al. [35], and the NW group populations harbored an additional coccoid microorganism with differences in distribution and frequency observed between the NW (A biotype) and Jatropha biotype populations. In both studies [35,39], the coccoid bacteria were sometimes observed in various states of degradation, indicating a possible mechanism of endosymbiont control by the host. Localization studies have since confirmed that pleomorphic *Portiera* is housed in the bacteriocytes, and secondary endosymbionts are located in the bacteriocytes or other host organs [13,40]. The early reports by Costa et al. [35,39] laid the groundwork for ultrastructural studies on the whitefly bacteriome, but few studies have followed up on this work. One exception, however, addressed the observed lack of cell wall in the primary endosymbiont, which is present in all other primary endosymbionts studied, providing evidence of such by electron microscopy [41].

Localization studies consistently place *Portiera* within the bacteriome; however, secondary endosymbionts exhibit more varied localization patterns [40]. Using fluorescence in situ hybridization (FISH) analysis, Gottlieb et al. [40] localized five of the seven known *B. tabaci* secondary endosymbiont genera in different life stages of MEAM1 and MED (Mediterranean) populations. *Hamiltonella* was identified in both MEAM1 and MED populations, and it was found in patches inhabiting only the bacteriome in all observed life stages (eggs, nymphs, and adults). Similarly, in the MED population *Arsenophonus* was located only in the bacteriome, but its distribution was more often surrounding the nuclei of bacteriocytes. Typically, *Arsenophonus* is observed exclusively in the bacteriome; however, Rana et al. [42] reported localization of this bacterium in the bacteriocytes, salivary glands, and midgut of *B. tabaci* Asia II. In eggs, nymphs, and adults of the MED population, *Wolbachia* was detected inside the bacteriome and concentrated along its circumference [40]. In some adult females, it was also found in the abdomen outside the bacteriome. *Cardinium* in the MED population and *Rickettsia* in MEAM1 and MED had wider distribution patterns than the other symbionts [40]. *Cardinium* was detected in the bacteriocytes in all stages, in higher concentrations in the abdomen and head in nymphs, in abdominal cells suspected by the authors to be fat cells in adult females, and widely distributed throughout the abdomen of adult males, excluding the rectal sac. *Rickettsia* interestingly had two very different distribution patterns, either strictly within the bacteriome concentrated around the circumference (confined phenotype) or, as previously observed [13], distributed throughout the body excluding the bacteriome (scattered phenotype) [40]. The specific phenotype was not correlated with the cryptic group nor collection location, and two populations (one MEAM1 and one MED) presented mixed phenotypes. Consistent with that observation, Shi et al. [43] found *Wolbachia* to have both confined and scattered phenotypes in *B. tabaci* AsiaII7. Similar localization patterns have been demonstrated for endosymbionts in other whitefly species, including *Arsenophonus* and *Hamiltonella* in *T. vaporariorum* [44] and *Rickettsia*, *Wolbachia*, and *Cardinium* in *T. vaporariorum* [45]. Thus far, bacteriocyte restriction has been observed for *Fritschea* in MEAM1 and NW2 [46] and *Hemipteriphilus* in China 1 [17]. Altogether, studies demonstrate that all secondary endosymbionts can co-occur with *Portiera* in the bacteriome and that *Hamiltonella*, *Fritschea*, and *Hemipteriphilus* are restricted to the bacteriocytes, while *Arsenophonus*, *Rickettsia*, *Wolbachia*, and *Cardinium* can occur in other whitefly tissues.

All *B. tabaci* endosymbionts are vertically (transovarial, maternal) transmitted [7]. Costa et al. [47] described the process of vertical transmission of the bacteriocytes. In *B. tabaci*, a single bacteriocyte cell is transferred to the plasma of an individual developing oocyte [47,48]. This process is different in other whitefly species, in which several cells are transferred [19]. When secondary endosymbionts locate within tissues other than the bacteriome (e.g., *Rickettsia*), the endosymbiont will temporarily colocalize with *Portiera* and any other endosymbionts within the bacteriocyte [44,49]. As the bacteriocyte enters and moves to the center of the egg, the endosymbiont will then exit the bacteriocyte and inhabit the egg cavity, while the bacteriome-inhabiting endosymbionts remain in the bacteriocyte. Some secondary endosymbionts are also capable of horizontal transmission, which can be plant-mediated via feeding (e.g., *Rickettsia* and *Wolbachia*; [50,51,52]) or mechanical via parasitoids [53,54]. Evidence of horizontal transmission of secondary endosymbionts of *B. tabaci* has been supported by phylogenetic incongruence between host and endosymbionts [24]. Ahmed et al. [24] demonstrated a lack of congruence between *Wolbachia* and *B. tabaci* and partial congruence for *Arsenophonus* and *Cardinium* with *B. tabaci*, suggesting that *Wolbachia* may be more adapted to horizontal transmission events. Plant-mediated horizontal transmission has been demonstrated for *Rickettsia*, which established in phloem cells after whitefly feeding and was acquired by *Rickettsia*-free whiteflies feeding on the same cotton leaf [50]. Host plant mediation of horizontal transmission was also found for *Wolbachia* [51] and between *B. tabaci* species (MEAM1 and MED) for *Rickettsia* [52]. Further, parasitoid-mediated horizontal transmission of *Wolbachia* and *Rickettsia* has been documented. *Wolbachia* was stably acquired by whiteflies after non-lethal probing by *Eretmocerus* sp. nr. *furuhashii* (Hymenoptera: Aphelinidae) contaminated with *Wolbachia* from whiteflies harboring the bacterium [53]. Whiteflies that acquired *Wolbachia* via the parasitoid had enhanced fitness. Other work has revealed that the *Rickettsia* and *Wolbachia* detected in field collections of *B. tabaci* nymphs and the emerging *Eretmocerus* parasitoids were identical haplotypes, suggesting that parasitoid-mediated horizontal transmission may be occurring between these species in the field [54].

### 2.3. Effects on Whitefly Fitness

Theoretically, secondary endosymbionts should provide some benefit to or increase the fitness of their hosts in return for the cost of harboring said endosymbiont. While studies have reported beneficial effects (e.g., [28,55,56,57,58,59,60]) and negative effects (e.g., [61,62,63]) of harboring certain endosymbionts, there remains a paucity of information about their fitness effects in *B. tabaci*, particularly given the species’ cryptic diversity and complex endosymbiont composition. To study the effects of specific endosymbionts on whitefly fitness, colonies that differ only in the presence or absence of a particular endosymbiont are ideally needed. In this respect, antibiotics have been used to obtain endosymbiont-free colonies. Endosymbiont elimination studies using antibiotic treatments of whiteflies have provided some clues as to the effects of endosymbionts on host fitness. Costa et al. [64] demonstrated that antibiotics have adverse effects on bacteriome size, number of bacteriome-inhabiting microorganisms, whitefly oviposition, adult growth, and progeny development. Antibiotic treatments of *B. tabaci* without identification of the specific bacteria reduced or eliminated demonstrated that antibiotics with different modes of action had differing effects on whitefly fitness. For example, antibiotics with action against bacterial protein synthesis (tetracycline and rifampicin) reduced host growth and development, while antibiotics with action against bacterial cell walls (penicillin, ampicillin, and lysozyme) did not have an observed effect on fitness [65]. Antibiotic selection is important because endosymbionts have differing susceptibilities [66,67,68]. Specific elimination of secondary endosymbionts via antibiotics has demonstrated positive or negative effects on host fitness [55,62,69,70,71,72]. However, some work has shown that selective, stable elimination of secondary endosymbionts may not be feasible for some populations [73]. Further, gut bacteria may have more influence on whitefly adaptation to host plants than maternally inherited endosymbionts [74]. This highlights a key question and potential issue with antibiotic elimination studies; that is, how many generations after the antibiotic treatment and elimination of a particular endosymbiont is it appropriate to assume that the gut bacteria have reestablished in endosymbiont populations? To perform reliable comparative studies considering questions of endosymbiont effects on fitness, it may be key that the gut microbiota is as similar in composition as possible between compared populations. Perhaps the results of some elimination studies have been more attributable to gut bacteria than to endosymbionts.

## 3. Whitefly Endosymbiont Evolution and Diversity

### 3.1. Bemisia tabaci Cryptic Species

The diversity and evolution of whiteflies has been a subject of extensive interest since the discovery of an exotic *B. tabaci* in the United States and the subsequent revelation of *B. tabaci* as a cryptic species composed of multiple sibling species [75,76,77]. *Bemisia tabaci* is reported to comprise over 40 species variants, which are differentiated by 3.5–4% divergence in the sequence of their mitochondrial cytochrome c oxidase I gene [37,38,78,79]. However, the exact bounds of distinct species are yet indeterminate, particularly because of the difficulty in proving species separation as defined by the biological species concept in field populations of *B. tabaci*. Despite the varied and evolving interpretations of *B. tabaci* species status over the years, many studies have demonstrated biological differences among cryptic *B. tabaci*, including, among others, host range [80], insecticide resistance [81], virus transmission [82], and composition of endosymbionts [39].

### 3.2. Primary Endosymbiont Ca. Portiera aleyrodidarum

The symbiosis of whiteflies with their primary endosymbiont *Portiera* is estimated to have begun with a single infection in a common ancestor of psyllids and whiteflies [10,83]. Phylogenetic studies reveal a high level of congruence between *Portiera* and host phylogenies [10], a pattern commonly seen in coevolved endosymbiont–insect host associations [84,85,86,87]. Co-speciation is further demonstrated by genomic decay in endosymbiont genomes, characterized particularly by the loss of genes unrelated to the essential functions the endosymbiont provides the host [88]. This pattern is especially pronounced in *Portiera* in *B. tabaci*, although unlike most highly reduced primary endosymbiont genomes, it has extended non-functional intergenic regions and tandem repeats [89,90]. Compared to *Portiera* in *T. vaporariorum*, the *B. tabaci* endosymbiont has notably lost several genes involved in DNA replication, recombination, and repair [89]. While the *Portiera* genomes of *T. vaporariorum* and two other Aleyrodids, *Aleurodicus dispersus* Russell and *Aleurodicus floccissimus* (Martin, Hernandez-Suarez, and Carnero), are syntenic, indicating evolutionary stasis, the genome of *B. tabaci Portiera* has undergone extensive rearrangements [90].

A counter-intuitive finding in many studies on the genomes of obligate intracellular endosymbionts of sap-feeding insects is the loss of genes involved in the biosynthesis of the 10 essential amino acids [91,92,93], the main function of the primary endosymbiont symbiosis with the insect host. In *B. tabaci* and other phytophagous hemipterans, several of these genes have been lost or pseudogenized, therefore requiring complements of those genes to be expressed by either the host or another symbiont [9,26,27,90,94,95]. Comparison of four *B. tabaci Portiera* genomes revealed that approximately half of their metabolism genes are involved in amino acid biosynthesis [26]. However, all genomes lacked 12 essential amino acid genes and have at least 2 pseudogenes in these pathways. Genomic and transcriptomic analysis showed that these deficiencies were compensated by host genes (including horizontally acquired genes of prokaryotic origin), secondary endosymbiont genes (*Hamiltonella* in MEAM1), and other bacterial genes [26]. Whitefly-*Portiera*-*Hamiltonella* pathway complementarity was demonstrated for *B. tabaci* MED, as well [27]. The genomic reduction of *Portiera* appears to be both compensated for and facilitated by genomic complementation in the host and other symbionts [26,27].

### 3.3. Secondary Endosymbionts

Secondary endosymbionts can be intricately involved in the essential metabolic processes of its insect host [26,27,28]. However, evidence of co-speciation is generally lacking for insect hosts and secondary endosymbionts. This is indicated by a lack of congruence in phylogenetic trees of host and secondary endosymbionts [7,10], by the wide variation and inconsistency in secondary endosymbiont-host associations (detailed for *B. tabaci* in a later section), and by evidence of multiple acquisitions of secondary endosymbionts [14,96]. The inconsistency of secondary endosymbiont–host associations is likely in part due to the nutritional and metabolic burden that they can place on the host and the extent to which these associations are parasitic or mutualistic [24,25].

*Bemisia tabaci* harbors the highest known diversity of secondary endosymbionts among phloem-feeding hemipterans [97]. Secondary symbiont infection patterns vary worldwide and regionally, with differences among populations and between native and invasive *B. tabaci* species. One of the earliest studies on endosymbiont composition in cryptic *B. tabaci* species demonstrated that US native NW populations had two distinct C-type (secondary) endosymbionts within their bacteriocytes whereas the invasive MEAM1 had only one [39]. This early report supported the hypothesis that the development of *B. tabaci* biotypes (now cryptic species) may have been facilitated by secondary endosymbionts and differences in their composition among the biotypes (see references in [39]). Since then, the prevalence, diversity, and phylogenetic relationships of endosymbionts in *B. tabaci* have been well documented from specific regions worldwide (e.g., [33,98,99,100,101,102,103,104,105,106,107]) and on a global scale among a variety of cryptic species (e.g., [11,12,79,108,109,110]).

Recently, using 16S rRNA sequences with associated geographical information retrieved from GenBank, a study on the global diversity of *B. tabaci* endosymbionts showed that three of the seven described genera of facultative endosymbionts are widespread in *B. tabaci* cryptic species [79]. *Rickettsia*, *Wolbachia*, and *Cardinium* were found in a wide diversity of *B. tabaci* species, including MEAM1, MED, and certain Asia, SSA (Sub-Saharan Africa), and NW cryptic species [79]. Of course, the specific endosymbionts present varied among and even within populations of these groups. According to this study, *Hamiltonella* and *Fritschea* were associated with only MEAM1, MED, and NW species, *Arsenophonus* was not associated with MEAM1, and *Hemipteriphilus* was associated with China 1 only. Further, phylogenetic analyses of the secondary endosymbionts demonstrated evidence of more than one genetic group for some endosymbionts (including 3 *Rickettsia*, 2 *Arsenophonus*, 2 *Wolbachia*, and 4 *Cardinium*) and only one group of *Hamiltonella*, which was conserved among MEAM1, MED, and NW species.

Another recent study, using PacBio sequencing of 16S rRNA bacterial genes from field populations of 23 cryptic species, also revealed wide genetic diversity in some secondary endosymbionts [110]. This study demonstrated 2 operational taxonomic units (OTUs) for *Rickettsia*, 5 OTUs for *Arsenophonus*, and 2 OTUs for *Wolbachia* [110]. One OTU each was found for *Hamiltonella*, *Cardinium*, *Fritschea*, *Hemipteriphilus*, and *Portiera*. Only one population of each *B. tabaci* cryptic species was sampled, however, Wang et al. [110] found *Hamiltonella* and, notably, *Hemipteriphilus* associated with more cryptic species than Kanakala and Ghanim [79] found. With the largely unpredictable nature of endosymbiont composition and diversity among *B. tabaci* cryptic species and populations, these studies highlight the improbability of secondary endosymbiont facilitated speciation of *B. tabaci*, the hypothesis proposed for cryptic *B. tabaci* early in the discovery of biotypes associated with different secondary symbionts.

## 4. Whitefly Endosymbiont-Plant Virus Interactions

### 4.1. Modes of Virus Transmission

For us, one of the more exciting avenues of research on hemipteran endosymbionts is the elucidation of the interactions of these bacteria with the plant viruses transmitted by their host. *Bemisia tabaci* transmits viruses in three distinct manners: non-persistently, semi-persistently, and in a persistent-circulative manner [5,111]. A fourth mode of biological transmission by hemipterans, persistent-propagative, is not known to occur in whiteflies. Based on the duration of infectivity after vector acquisition and virus route of movement within the vector, viruses are grouped as circulative or non-circulative [111]. Non-persistent and semi-persistent, both non-circulative transmission types, describe interactions of the virus with its insect vector that are characterized by localization and retention of the virus within the vector’s stylets or foregut before transmission. Whereas, persistent-circulative and persistent-propagative, both circulative modes of transmission, are characterized by virus acquisition into the vector’s hemolymph via the filter chamber, midgut, or hindgut and circulation of the virus to the salivary glands for subsequent transmission, with propagative viruses having the additional capability to replicate in the vector. *Bemisia tabaci* is notorious for its persistent-circulative transmission of begomoviruses (family *Geminiviridae*), for which this species is the only demonstrated vector. *Bemisia tabaci* also transmits viruses in the genera *Crinivirus* (family *Closteroviridae*), *Ipomovius* (family *Potyviridae*), *Carlavirus* (family *Betaflexiviridae*), and *Torradovirus* (family *Secoviridae*) in a non-circulative manner [4,5,111,112,113,114]. The manner by which plant viruses are transmitted may inherently determine, at least in part, whether or not there is an interaction between vector endosymbionts and their metabolites and the insect-transmitted virus. That is, circulative viruses may be more likely to have such interactions than non-circulative viruses, which are more transiently associated with the vector and are not acquired into the hemolymph.

### 4.2. Endosymbiont Expressed GroEL

The first reported study to suggest a possible role of endosymbionts in transmission of plant viruses was van den Heuvel et al. [115]. This study demonstrated that potato leafroll virus (genus *Polerovirus*, family *Luteoviridae*) binds to symbionin (homolog to *Escherichia coli* GroEL chaperonin protein; [116]) expressed by the primary endosymbiont of *Myzus persicae* (Sulzer) (Hemiptera: Aphididae). Additional studies with aphids and circulative luteoviruses further demonstrated this interaction, suggesting it as a means of virus protection from degradation in the hemolymph by the vector immune system and emphasizing the probable role of endosymbiont expressed GroEL-like protein in the virus transmission process [117,118,119].

The differences between luteoviruses and geminiviruses are many, but begomoviruses share a similar persistent-circulative mode of transmission, with a key barrier to transmission being persistence in the vector hemolymph subsequent to passing through the gut tissue [120]. While it was the primary aphid endosymbiont, *Buchnera*, that was demonstrated to express the GroEL-like chaperonin in *M. persicae*, and *Buchnera* is unrelated to the primary endosymbiont of whiteflies, a secondary endosymbiont related to *Buchnera* is present in some whiteflies [36]. This led researchers to investigate the possibility of whitefly endosymbiont GroEL-like chaperonin (previously symbionin; hereafter simply referred to as GroEL) expression and interaction with the begomovirus tomato yellow leaf curl virus (TYLCV). Morin et al. [18] demonstrated that GroEL was expressed by the coccoid secondary endosymbiont (C-type; [35,39]) of *B. tabaci* B biotype (MEAM1), that TYLCV showed an affinity for GroEL, and that TYLCV transmission was significantly (>80%) reduced with the introduction of anti-GroEL antiserum. Both insect transmissible (TYLCV-Israel) and non-transmissible (abutilon mosaic virus from Israel; AbMV-Is) begomoviruses interacted with GroEL in vitro, indicating the inability of whitefly transmission of AbMV-Is (and lack of AbMV-Is detection in whitefly hemolymph) is likely due to an inability to cross the gut epithelial barrier rather than a lack of affinity to *B. tabaci* GroEL [121]. Similarly, several luteoviruses bind to the GroEL chaperonins produced by both vector and non-vector aphid species in vitro [122,123]. Altogether, these studies show the importance of GroEL–begomovirus/–luteovirus interactions for virus protection in the vector hemolymph, where insect defenses may otherwise degrade the foreign invader, as one component of transmission competency, but other factors ultimately determine virus-vector transmission specificity.

### 4.3. Hamiltonella

Since the early work on GroEL–begomovirus interactions, the number of secondary endosymbionts discovered in whiteflies has increased from the two previously described C-types in *B. tabaci* MEAM1 and NW [35,39] to now seven different genera identified in different combinations and localizations in *B. tabaci* cryptic species. Studies to determine which secondary endosymbionts and to what degree are responsible for expression of GroEL correlated with virus transmission began with looking at populations of *B. tabaci* MEAM1 and MED from Israel. At that time, *Rickettsia* was known to occur in the Israeli MEAM1 and MED, *Hamiltonella* was identified in MEAM1 only, and *Wolbachia* and *Arsenophonus* were identified in MED only [13,98,124]. While all secondary endosymbionts, as well as the primary symbiont *Portiera*, expressed GroEL in the hemolymph of both MEAM1 and MED, only GroEL expressed by *Hamiltonella* in the MEAM1 biotype interacted with TYLCV resulting in transmission (Table 2; [124]). MED transmitted TYLCV at a significantly lower rate, and evidence of TYLCV-GroEL interaction in vitro was not found. Early on then, *Hamiltonella* was implicated in assisting begomovirus transmission via protection from the insect immune response, and other studies have emphasized this relationship and correlated this interaction with increased virus transmission by other *B. tabaci* cryptic species (MED in China and Brazil, respectively; [125,126]). In a comparison of the native NW2 *B. tabaci* with exotic MEAM1 and MED in Brazil, the *Hamiltonella* GroEL chaperone sequence of NW2 was found to be deficient in three amino acids [127]. Given the interaction of *Hamiltonella* GroEL with whitefly transmitted viruses, this was suggested as one possible factor affecting differential transmission of viruses, including a begomovirus and non-circulative crinivirus and carlavirus, by NW2 and MEAM1 in Brazil [127,128]. Interestingly, Bello et al. [126] demonstrated that MED *B. tabaci* with higher frequencies of *Hamiltonella* transmitted the non-circulative viruses cowpea mild mottle virus (genus *Carlavirus*) and tomato chlorosis virus (genus *Crinivirus*) at higher rates. Therefore, the *Hamiltonella*-plant virus interaction is not restricted to *B. tabaci* MEAM1, and it has implications in the epidemiology of begomoviruses worldwide as well as some non-circulative viruses.

### 4.4. Rickettsia

*Bemisia tabaci*-harbored *Rickettsia* has also been implicated in virus interactions within the vector (Table 2). Isoline colonies of *Rickettsia*+ MEAM1 *B. tabaci*, which also harbored *Hamiltonella*, acquired TYLCV at a higher rate and transmitted the virus with greater efficiency [129]. When whiteflies were highly infected with *Rickettsia* localized in the midgut, TYLCV virions were detected at higher titers in the filter chamber (along with the anterior midgut, where begomovirus virions cross into the hemolymph; [132]), likely where higher concentrations of the virus allowed increased translocation into the hemolymph. Interestingly, while vector infection with *Rickettsia* enabled increased virus transmission, the dynamic between the endosymbiont and virus appeared somewhat antagonistic with their spatial segregation in the gut and decreased levels of *Rickettsia* as virus was acquired [129]. Another interesting finding was that *Hamiltonella* levels increased in the *Rickettsia*– strain after acquisition of TYLCV but remained the same in the *Rickettsia*+ strain. While virus acquisition by the *Rickettsia*– strain was lower, perhaps *Hamiltonella* increased to facilitate transmission of those virions that crossed into the hemolymph. Further research demonstrated that acquisition of TYLCV induced an activation of immune system gene expression in *Rickettsia*– MEAM1 *B. tabaci* but had the opposite effect and induced a down-regulation of immunity-related genes in *Rickettsia*+ *B. tabaci* [130]. More recently, presence of the newly discovered species of maternally inherited *Rickettsia*, *Candidatus* Rickettsia Torix *Bemisia tabaci* (RiTBt), in *B. tabaci* Asia II 7 [23,110] slightly increased virus titer in the vector and transmission of cotton leaf curl Multan virus (genus *Begomovirus*, family *Geminiviridae*) after a prolonged acquisition access period [131]. RiTBt was localized to the bacteriocyte only, even after virus acquisition, and was therefore hypothesized to affect virus transmission via secretory proteins.

### 4.5. Other Secondary Endosymbionts

Fewer studies have focused on the roles of the other secondary endosymbionts in virus transmission. While, previously, *Hamiltonella* GroEL was the only endosymbiont-expressed GroEL thought to interact with begomoviruses in *B. tabaci*, Rana et al. [42] demonstrated that the secondary endosymbiont *Arsenophonus* of an Asia II population devoid of *Hamiltonella* expressed GroEL which interacted with cotton leaf curl virus (genus *Begomovirus*, family *Geminiviridae*) in vitro and in vivo (Table 2). Unlike *Hamiltonella* and previous reports of *Arsenophonus* restriction to the bacteriocyte, *Arsenophonus* in this population of *B. tabaci* was also found in the salivary glands and midgut, two organs involved in the transmission process [42]. Another report on the effect of *Arsenophonus* on virus transmission found that fitness of an African SSA1-SG3 *B. tabaci* (cassava whitefly) population was negatively impacted by harboring *Arsenophonus* and *Rickettsia* (AR+) and that acquisition and retention of East African cassava mosaic virus-Uganda variant was lower in AR+ *B. tabaci* [63]. We are not aware of any described interactions of *Portiera* or secondary endosymbionts *Wolbachia*, *Cardinium*, *Fritschea*, and *Hemipteriphilus* with plant viruses in *B. tabaci* transmission.

## 5. Conclusions

Tri-trophic vector-endosymbiont-plant and vector-endosymbiont-virus interactions are indeed complex considering the cryptic *B. tabaci* complex with its numerous sibling species, several secondary endosymbionts inhabiting their hosts in different combinations, and transmission of plant viruses. Understanding all levels involved (vector, bacterial, and viral) and their activities in host interactions and virus transmission processes are key to developing novel pest management strategies targeting endosymbionts or their products. With the application of high throughput sequencing technology to the study of whitefly metagenomes, the issue of false negatives in PCR-based endosymbiont detection has been minimized, allowing a more complete picture of the microorganisms involved in the whitefly holobiont. While endosymbionts play important roles in whitefly–host plant interactions, other symbiotic bacteria, including gut bacteria, likely influence these interactions. It is central then to accurate research on endosymbiont roles in whitefly fitness and whitefly–host plant and -virus interactions to demonstrate the establishment of comparative populations whose microbiome compositions are, ideally, identical. Using antibiotic treatments to establish endosymbiont-free populations disrupts the microbiome; therefore, efforts to reestablish the microbiota (excluding the endosymbiont of interest) should be considered. Selective elimination of endosymbionts which are generally not fixed in populations, such as *Rickettsia* and *Wolbachia*, is possible without antibiotics; however, those which generally are fixed, such as *Hamiltonella* and *Arsenophonus*, present more of a challenge.

While many questions regarding the effects of particular endosymbionts on whitefly behavior and fitness remain, the same is true for endosymbiont influence in virus transmission. Studies have demonstrated the involvement of endosymbiont expressed proteins, such as GroEL, in the virus transmission process. Given this interaction and that the GroEL chaperone is highly expressed by intracellular endosymbionts, the question of coevolved relationships arises. Perhaps the relationship is one of chance, or maybe the protection of begomovirus virions against the vector immune system by secondary endosymbiont GroEL is a coevolved relationship which benefits both whitefly/endosymbiont and virus. With improvements on the resolution of in vivo endosymbiont transcriptomes, studying the effects of virus acquisition on endosymbiont gene expression using comparative transcriptomics could address this question. Whitefly gene expression [133] and behavior [134,135] are altered after feeding on TYLCV infected tomatoes. These observations along with the demonstrated endosymbiont–begomovirus interactions raise questions about how the endosymbionts are affected by or involved in these processes and if they may be targeted to reduce or prevent virus transmission. Given the evidence of such multitrophic interactions and recent advancements in genome-based biotechnologies, management of whiteflies and whitefly transmitted viruses via endosymbiont-targeted manipulation is possible. For example, endosymbiont-mediated RNA interference (RNAi) has been demonstrated for control of the western flower thrips (WFT) [136]. In that study, a cultivable symbiotic gut bacterium was isolated from the WFT and transformed with a dsRNA expression cassette targeting the WFT alpha-tubulin gene. Re-introduction of the gut endosymbiont and colonization in its host resulted in heritable target knockdown and high mortality in first instars [136]. While direct manipulation of maternally inherited primary or secondary endosymbionts presents more of a challenge due to their fastidious nature, endosymbiont genes that are involved in production of essential amino acids or carotenoids [21,26,27] or in virus transmission [18,121] may be targeted for specific *B. tabaci* or virus management. Disentangling these multitrophic relationships should unlock new ideas, leading to needed advancements in managing this global pest.

## Figures and Tables

**Table 1:** Taxonomic, biological, and genomic characteristics of the primary and secondary endosymbionts of cryptic *Bemisia tabaci*. Genome information was obtained from NCBI (https://www.ncbi.nlm.nih.gov/) from the earliest and most complete genome assembly for each endosymbiont where available. When the sequence publication reference was not given, the BioProject accession number was provided.

Endosymbiont	Symbiosis	Phylum	Order	*Bemisia tabaci* Species	Localization	Genome Size (Mb)	Assembly Level	Protein Coding Genes	Assembly Accession #	Reference
*Ca.* Portiera aleyrodiarum	Obligate	Proteobacteria	Oceanospirillales	MEAM1	Bacteriome	0.36	Complete	258	GCA_000292685	[21]
*Ca.* Hamiltonella defensa	Facultative	Proteobacteria	Enterobacterales	MEAM1	Bacteriome	1.74	Complete	1466	GCA_002285855	[9]
*Ca.* Arsenophonus	Facultative	Proteobacteria	Enterobacterales	Asia II 3	Bacteriome, salivary glands, midgut	2.33	Contig	1691	GCA_004118055	PRJNA327006
*Ca.* Cardinium hertigii	Facultative	Bacteroidetes	Cytophagales	China	Bacteriome, abdomen, head	1.00	Scaffold	768	GCA_004300865	PRJNA299728
*Ca.* Fritschea bemisiae	Facultative	Chlamydiae	Parachlamydiales	-	Bacteriome	-	-	-	-	-
*Ca.* Hemipteriphilus asiaticus	Facultative	Proteobacteria	Rickettsiales	-	Bacteriome	-	-	-	-	-
*Ca.* Rickettsia Bellii	Facultative	Proteobacteria	Rickettsiales	MEAM1	Bacteriome or external to bacteriome	1.38	Complete	1278	GCA_002285905	[9]
*Ca.* Rickettsia Torix	Facultative	Proteobacteria	Rickettsiales	Asia II 7	Bacteriome, midgut, salivary gland, ovaries, testes	1.12	Scaffold	1301	GCA_013435745	[23]
*Ca.* Wolbachia	Facultative	Proteobacteria	Rickettsiales	China 1	Bacteriome and/or external to bacteriome	1.31	Chromosome	979	GCA_003999585	PRJNA327485

**Table 2:** Summary of the interactions of *Bemisia tabaci* endosymbionts with plant viruses demonstrated by in vitro or in vivo experiments.

*Bemisia tabaci* species	Endosymbiont(s)	Endosymbiont Product	Virus Names ^a^	Effect on Transmission	Reference
MEAM1	Undetermined	GroEL chaperone	Tomato yellow leaf curl virus	Facilitatory	[18,121]
MEAM1	Undetermined	GroEL chaperone	Abutilon mosaic virus ^b^	~	[121]
MEAM1	*Hamiltonella*	GroEL chaperone	Tomato yellow leaf curl virus	Facilitatory	[124]
MED	*Hamiltonella*	Unspecified	Tomato yellow leaf curl virus	Facilitatory	[125]
MED	*Hamiltonella*	Unspecified	Cowpea mild mottle virus, bean golden mosaic virus, tomato chlorosis virus	Increased transmission	[126]
MEAM1	*Rickettsia*	Unspecified	Tomato yellow leaf curl virus	Increased acquisition and transmission	[129]
MEAM1	*Rickettsia*	Unspecified	Tomato yellow leaf curl virus	Down-regulation of whitefly immunity genes	[130]
Asia II 1	*Rickettsia* (Torix)	Hypothesized secretory proteins	Cotton leaf curl Multan virus	Increased virus titer and transmission	[131]
Asia II	*Arsenophonus*	GroEL chaperone	Cotton leaf curl virus	Undetermined	[42]
SSA1-SG3	*Arsenophonus*, *Rickettsia*	Undetermined	East African cassava mosaic virus-Uganda	Reduced acquisition and retention	[63]

^a^ All viruses are members of genus *Begomovirus* (family *Geminiviridae*) except cowpea mild mottle virus (genus *Carlavirus*, family *Betaflexiviridae*) and tomato chlorosis virus (genus *Crinivirus*, family *Closteroviridae*).^b^ Abutilon mosaic virus is non-transmissible by *B. tabaci*.
